# New Case of Spinocerebellar Ataxia, Autosomal Recessive 4, Due to *VPS13D* Variants

**DOI:** 10.3390/ijms25105127

**Published:** 2024-05-08

**Authors:** Denis Kistol, Polina Tsygankova, Fatima Bostanova, Maria Orlova, Ekaterina Zakharova

**Affiliations:** Research Centre for Medical Genetics, 115522 Moscow, Russia

**Keywords:** *VPS13D*, spinocerebellar ataxia, Leigh syndrome, mitochondrial dysfunction

## Abstract

Movement disorders such as bradykinesia, tremor, dystonia, chorea, and myoclonus most often arise in several neurodegenerative diseases with basal ganglia and white matter involvement. While the pathophysiology of these disorders remains incompletely understood, dysfunction of the basal ganglia and related brain regions is often implicated. The *VPS13D* gene, part of the *VPS13* family, has emerged as a crucial player in neurological pathology, implicated in diverse phenotypes ranging from movement disorders to Leigh syndrome. We present a clinical case of *VPS13D*-associated disease with two variants in the *VPS13D* gene in an adult female. This case contributes to our evolving understanding of *VPS13D*-related diseases and underscores the importance of genetic screening in diagnosing and managing such conditions.

## 1. Introduction

Movement disorders most often arise from several neurodegenerative or structural diseases of the basal ganglia and internal capsule. They may also be part of the clinical manifestations of systemic metabolic disorders as the initial feature, complicating their course [1]. Movement disorders can be divided into two main categories: hyperkinetic disorders, which include dystonia, chorea, tremor, and myoclonus, and hypokinetic movements, such as bradykinesia. The pathophysiology of movement disorders is not yet fully understood, but most abnormal movements are associated with dysfunction of the basal ganglia or their interconnected brain regions, such as the thalamus, cerebellum, and sensorimotor cortex [2].

The *VPS13D* gene (OMIM *608877; NM_015378.4), also known as the vacuolar protein sorting 13 homolog D gene, is part of the *VPS13* family of widely expressed genes encoding four proteins highly conserved in eukaryotic cells (*VPS13A-D*) [3]. The *VPS13D* gene is located on chromosome 1p36 and spans about 280 kb of genomic DNA, with 70 exons [4]. VPS13D is a bulk lipid transporter of 4388 amino acid residues located at membrane contact sites and is one of the lipid transfer proteins (LTPs) containing the so-called repeating β-groove (RBG) rod-like structure. In protein structure, N-terminal and C-terminal regions can be recognized. The N-terminal region of the VPS13D protein displays a Chorein N domain (also known as the VPS13 N-terminal domain) and two phenylalanines in an acidic amino acid track tract (FFAT), which binds VAMP-associated proteins (VAP), tethering the endoplasmic reticulum (ER). The RBG rod is flanked at the C-terminal region by three folded modules that mediate its interaction with membranes. These are an arcuate protrusion of the rod core called the Vps13 adapter binding region (VAB); a bundle of four amphipathic α-helices following the aberrant pollen transmission 1 (APT1) portion of the RBG stem, called ATG2-C because of its homology to the corresponding region in ATG2; and a C-terminal pleckstrin homology (PH) domain connected to ATG2-C by a short conserved flexible loop. Uniquely, VPS13D is the only homologue that expresses the ubiquitin-associated (UBA) domain that is conserved from fly to human [5]. *VPS13D* is an important gene required for autophagy, changes in mitochondrial size, and mitochondrial clearance and LoF (loss-of-function) mutations result in enlarged spherical mitochondria that accumulate in the perinuclear region and often break [6].

Pathogenic variants in the *VPS13D* gene have been consistently reported in patients with variable neurological phenotypes, typically with a predominance of movement disorders [2,7]. The Online Mendelian Inheritance in Man database (OMIM) contains only one described phenotype of the *VPS13D* gene dysfunction: spinocerebellar ataxia, autosomal recessive 4 (SCAR4; OMIM #607317) (for date 24 April 2024). However, the literature also presents patients with more variable clinical symptoms, including spastic paraplegia, spastic ataxia, chorea, dystonia, tremor, and seizures. In two publications, patients with pathogenic variants in the *VPS13D* gene were assigned to Leigh syndrome (LS; OMIM #256000), a frequent mitochondrial pathology of childhood [8,9].

Currently, about 33 patients with pathogenic variants in the *VPS13D* gene have been described [10].

Herein, we present a new clinical case of *VPS13D*-associated disease with two single-nucleotide variants in the *VPS13D* gene in an adult female.

## 2. Case Presentation

**Figure 1:**
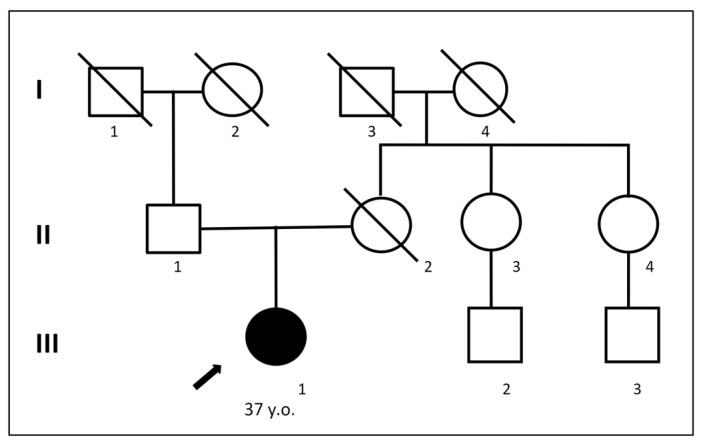
Pedigree of the *VPS13D* patient from our study.

**Figure 2:**
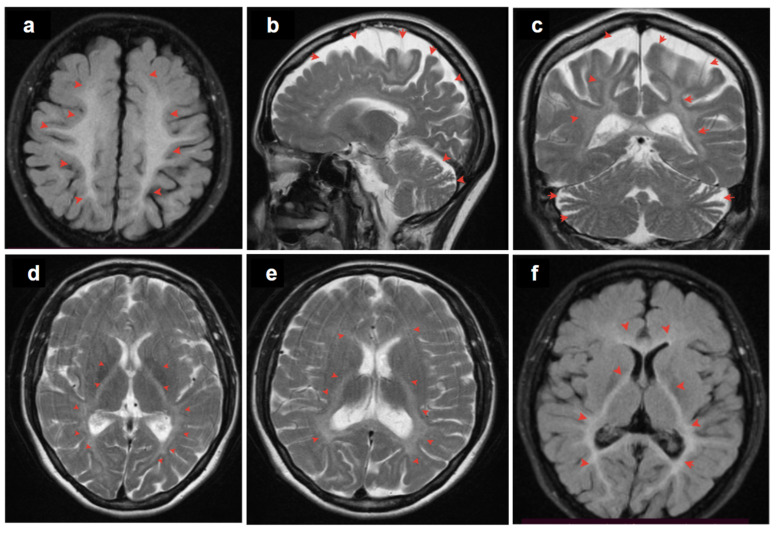
MRI images of the brain: diffuse, bilateral hyperintensities in the subcortical white matter (**a**); cortical and cerebellar atrophy (**b**,**c**); coronal view of the posterior subcortical and periventricular T2 hyperintensities (**c**); posterior limb of the internal capsule and periventricular areas (**d**–**f**). Red arrows point to the affected regions.

The patient was a 37-year-old woman, the only daughter of non-consanguineous parents (Figure 1). Her early development was normal. However, at the age of 35, dysarthria appeared, characterized by slow and slurred speech along with hand tremors. The condition progressed over the course of a year. Brain MRI revealed leukoencephalopathy with cortical and cerebellar atrophy (Figure 2).

Upon examination at 37 years of age, she exhibited horizontal nystagmus, dysarthria, hyperreflexia, upper Rossolimo reflex, Jacobson-Lask reflex, instability on Romberg’s test, postural tremor, dysdiadochokinesia, intention tremor, and clonus.

Clinical Exome Sequencing (CES) analysis revealed two nucleotide variants in the *VPS13D* gene: a heterozygous nonsense variant c.9388C>T, p.(Arg3130Ter), described earlier (ClinVar variation ID 3016121), and a novel heterozygous missense variant c.9679G>T, leading to amino acid substitution p.(Gly3227Trp) in the VPS13D protein. Both variants were validated by Sanger sequencing.

The transposition of the c.9388C>T, p.(Arg3130Ter), and c.9679G>T, p.(Gly3227Trp) variants in the *VPS13D* gene was confirmed using the molecular cloning method as parents were not available for segregation analysis. According to the ACMG criteria, the variant c.9388C>T, p.(Arg3130Ter) has been classified as pathogenic (PVS1, PM2, PP5), and the variant c.9679G>T, p.(Gly3227Trp) should be considered as likely pathogenic (PM2, PP3, PP2, PM3 moderate).

The identified missense variant, c.9679G>T, p.(Gly3227Trp), is located in the VPS13 adaptor binding domain, previously known as SHR-BD, found in the VPS13 family. Because VPS13 is conserved in eukaryotes, much of our understanding of these proteins arises from studies performed in the budding yeast, Saccharomyces cerevisiae, which has a single Vps13 protein unlike the human VPS13A-D family. This domain interacts with membrane-specific adaptor proteins such as Ypt35 (which recruits Vps13 to endosomal and vacuolar membranes), Spo71 (which recruits Vps13 to the prospore membrane during meiosis), and the mitochondrial membrane protein Mcp1 (which targets Vps13 at mitochondria) in yeast to be recruited to different membranes [11].

**Figure 3:**
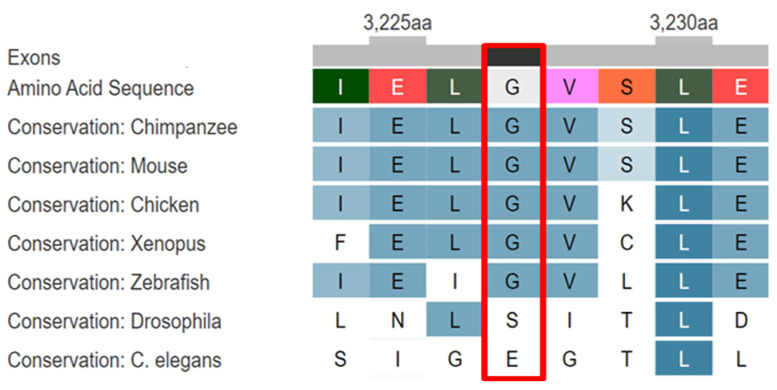
Amino acid alignment of the Vps13 adaptor binding domain from different species, with cutoffs for identified variants. The alignment was adapted from DECIPHER database (https://www.deciphergenomics.org/, accessed on 24 April 2024). The red rectangle highlights the identified amino acid substitution from the study.

Bioinformatics analysis by AlphaMissense tool (https://alphamissense.hegelab.org/, accessed on 24 April 2024) indicates that the substitution of glycine for tryptophan at position 3227 has a high-pass pathogenicity score of 0.975 and is assessed as likely pathogenic. The substitution occurs in a moderately conserved region (Figure 3) and also in the functional domain of the protein. It can be postulated that the substitution of threonine for isoleucine, due to the greater hydrophobicity of the former, results in a disruption of the spatial and hydrophobic interactions within the protein and a change in the functional activity of the protein.

## 3. Discussion

**Figure 4:**
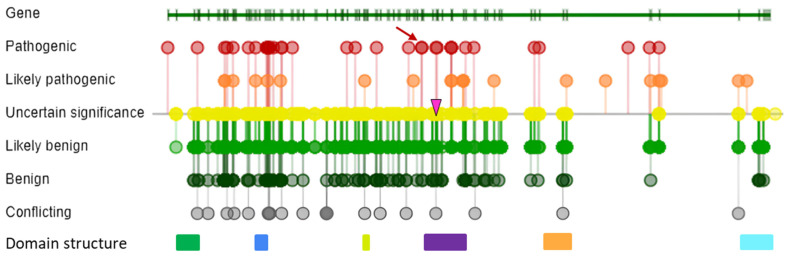
A schematic representation of the *VPS13D* gene structure and of the domain structure (colored rectangle) of the VPS13D protein (green—chorein N-terminal domain, blue—RBD rod-like structure, light green—UBA domain, purple—VAB domain, orange—DH-like domain, and turquoise—C-terminal domain). The location of nucleotide variants in the gene, as identified by the ClinVar database (https://www.ncbi.nlm.nih.gov/clinvar/, accessed on 24 April 2024), is indicated by colored dots (red—pathogenic; orange—likely pathogenic, yellow—variant uncertain significance, likely benign—light green, green—benign); variants identified in the present study are indicated: a nonsense variant, c.9388C>T, p.(Arg3130Ter), in exon 46 (red arrow) and a missense variant, c.9679G>T, p.(Gly3227Trp), in exon 47 (pink triangle). Abbreviations: UBA—ubiquitin-associated, VAB—Vps13 adaptor binding, RBG—repeating β-groove.

The spectrum of pathogenic variants in *VPS13D*, initially described in 2018 by Gauthier et al., continues to expand. To date, 39 variants have been described in the Human Gene Mutation Database (HGMD version 2022.1), including 25 missense, eight nonsense, three frameshift, two gross, and one splice variant. In addition, the ClinVar database (25 April 2024) contains 52 pathogenic or likely pathogenic variants, with 21 being nonsense and 16 being frameshift variants (Figure 4).

The most severe phenotype of *VPS13D*-associated disorders is Leigh-like syndrome, which manifests in childhood. Cases with a Leigh-like phenotype were previously described by Gauthier et al. (2018), Lee et al. (2020), and Kistol et al. (2023) [2,8,9], totaling five cases. In our previous study, we described a 6-year-old boy, exhibited developmental delay, ataxia, epileptic seizures, and neuroimaging findings indicative of basal ganglia involvement. His clinical presentation, marked by hypotonia and increased blood lactate, initially inclined clinicians to consider primary mitochondrial diseases. However, the entirety of his clinical picture, including ataxic gait and dysmorphic features, aligns with the spectrum of *VPS13D-*related phenotypes. Despite association with Leigh-like cases, *VPS13D* does not directly influence OXPHOS. Nonetheless, its essential role in autophagy, mitochondrial size, and clearance could manifest in “mitochondrial” phenotypes [6].

The patient described here, a 37-year-old woman, experienced symptoms that manifested in her adulthood, such as dysarthria and hand tremors. Despite variations in age of onset and clinical features, the patient’s presentation shares similarities with patients with early manifestations, particularly regarding neurological manifestations like dysarthria, tremors, and hyperreflexia. Leukoencephalopathy with internal capsule lesions has been described earlier in *VPS13D* patients but is not typical for all of them (3/32 patients). The most frequent brain MRI abnormality is cerebellar atrophy (11/20) [10].

Novel cases illustrate the diverse and progressive nature of *VPS13D*-related disorders, which can emerge in children and adults with varying severity. Symptoms associated with *VPS13D*-related disorders span a broad spectrum of severity and variability, encompassing cerebellar ataxia, other movement disorder manifestations such as dystonia, chorea, and tremor, cognitive impairment, and spastic paraplegia. Additionally, reports have included chorioretinal dystrophy, seizures, hearing loss, and microcephaly [2,7,12]. Prior studies have observed symmetrical T2-weighted hyperintensities affecting the basal ganglia and brainstem, along with multifocal white matter involvement in specific patients. Furthermore, mild to moderate sensorimotor axonal polyneuropathy has been documented.

The intricate clinical presentations and evolving understanding of *VPS13D*-related disorders underscore the ongoing need for multidisciplinary approaches to diagnosis and management, as well as further research into the underlying pathophysiological mechanisms.

## 4. Materials and Methods

### 4.1. Editorial Policies and Ethical Considerations

This work was carried out in accordance with The Code of Ethics of the World Medical Association Declaration of Helsinki for experiments involving humans. The study was approved by the local ethics committee of the Federal State Budgetary Institution “Research Centre for Medical Genetics”. The approval number is 2015–5/3. Date 3 May 2015. Informed consent was obtained from all patients.

### 4.2. DNA and RNA Extraction, and Sanger Sequencing

Genomic DNA was extracted from blood samples with the use of the QiaAMP DNA-mini kit (Qiagen, Germantown, MD, USA), following the manufacturer’s protocol.

Total RNA was isolated from whole blood cells using the Leukocyte RNA Purification Plus Kit (Norgene, Thorold, ON, Canada). The first strand of cDNA was synthesized using ImProm-II™ Reverse Transcriptase (Promega, Madison, WI, USA) and oligo(dT) primers.

Sanger sequencing was performed using an ABI Prism 3500XL (Thermo Fisher Scientific, Waltham, MA, USA), following the manufacturer’s protocol. Genetic variants are named according to the GRCh37 (hg19) genome assembly.

### 4.3. CES

Clinical exome sequencing (CES) was performed with the TruSeq DNA PCR-Free sample preparation kit on a NovaSeq 6000 (Illumina, San Diego, CA, USA).

Bioinformatic pipeline: sequence reads were aligned to the human reference genome GRCh37 (hg19) using Burrows-Wheeler Aligner (http://bio-bwa.sourceforge.net/, accessed on 24 April 2024). Single-nucleotide variants and small insertions and deletions (indels) were called with the Strelka2 Small Variant Caller (https://github.com/Illumina/strelka/, accessed on 24 April 2024) and the Genome Analysis Toolkit v.4 (https://gatk.broadinstitute.org/, accessed on 24 April 2024). The reported variants were annotated with their genomic coordinates, allele frequency (gnomAD database, http://gnomad.broadinstitute.org/, accessed on 24 April 2024), functional consequence and Impact level on the gene product using SnpEff v5 (http://pcingola.github.io/SnpEff/, accessed on 24 April 2024). Variants were prioritized by the consensus score of the set of bioinformatic tools that predict the pathogenicity of the variant and its deleterious effect on proteins (SIFT, SIFT4G, Polyphen2, MutationAssessor, FATHMM, PROVEAN, DEOGEN2, LRT, PrimateAI, MetaSVM, MetaLR, SpliceAI, MMsplice, SPiP, and Spidex). Data analysis was performed with the in-home NGSData-Genome interface.

Variants were named according to the HGVS nomenclature and validated using VariantValidator (https://variantvalidator.org/, accessed on 24 April 2024). The novel variants were classified according to ACMG recommendations [13].

### 4.4. Cis-/Trans-Position Assay

The 615 bp cDNA fragment of *VPS13D* exons 46–47, containing both studied variants, was cloned into the plasmid vector and transformed into competent *E. coli* cells. Plasmid DNA from single bacterial colonies was amplified with plasmid-specific primers and Sanger sequenced.

## 5. Conclusions

The presented case contributes to our understanding of the clinical and molecular spectrum of *VPS13D*-associated disorders, emphasizing the need for comprehensive evaluation and genetic testing in patients presenting with leukoencephalopathy and neurodegenerative phenotypes. This highlights the importance of recognizing these phenotypes, especially in patients presenting with childhood-onset ataxia-plus syndrome or suspected hereditary spastic paraplegia. It is of particular importance to emphasize the clinical phenotype of SCAR4 in such cases, as it represents a distinct yet interconnected entity within this spectrum. It is worth noting that rare cases of LS may be attributed to genes not directly implicated in OXPHOS function, such as those associated with *VPS13D*-related ataxia phenotypes, serving as a notable illustration of LS phenocopy. Further research is warranted to elucidate the underlying pathophysiological mechanisms and identify potential therapeutic targets for these complex and challenging conditions.

## Data Availability

Data are contained within the article.

## References

[1] Pedroso J.L., Barsottini O.G., Espay A.J. (2019). Movement Disorders in Metabolic Disorders. Curr. Neurol. Neurosci. Rep..

[2] Gauthier J., Meijer I.A., Lessel D., Mencacci N.E., Krainc D., Hempel M., Tsiakas K., Prokisch H., Rossignol E., Helm M.H. (2018). Recessive mutations in *VPS13D* cause childhood onset movement disorders. Ann. Neurol..

[3] Rzepnikowska W., Flis K., Muñoz-Braceras S., Menezes R., Escalante R., Zoladek T. (2017). Yeast and other lower eukaryotic organisms for studies of Vps13 proteins in health and disease. Traffic.

[4] Velayos-Baeza A., Vettori A., Copley R.R., Dobson-Stone C., Monaco A.P. (2004). Analysis of the human VPS13 gene family. Genomics.

[5] Hanna M., Guillén-Samander A., De Camilli P. (2023). RBG Motif Bridge-Like Lipid Transport Proteins: Structure, Functions, and Open Questions. Annu. Rev. Cell Dev. Biol..

[6] Anding A.L., Wang C., Chang T.-K., Sliter D.A., Powers C.M., Hofmann K., Youle R.J., Baehrecke E.H. (2018). Vps13D Encodes a Ubiquitin-Binding Protein that Is Required for the Regulation of Mitochondrial Size and Clearance. Curr. Biol..

[7] Koh K., Ishiura H., Shimazaki H., Tsutsumiuchi M., Ichinose Y., Nan H., Hamada S., Ohtsuka T., Tsuji S., Takiyama Y. (2020). *VPS13D*-related disorders presenting as a pure and complicated form of hereditary spastic paraplegia. Mol. Genet. Genom. Med..

[8] Lee J.S., Yoo T., Lee M., Lee Y., Jeon E., Kim S.Y., Lim B.C., Kim K.J., Choi M., Chae J. (2020). Genetic heterogeneity in Leigh syndrome: Highlighting treatable and novel genetic causes. Clin. Genet..

[9] Kistol D., Tsygankova P., Krylova T., Bychkov I., Itkis Y., Nikolaeva E., Mikhailova S., Sumina M., Pechatnikova N., Kurbatov S. (2023). Leigh Syndrome: Spectrum of Molecular Defects and Clinical Features in Russia. Int. J. Mol. Sci..

[10] Pauly M.G., Brüggemann N., Efthymiou S., Grözinger A., Diaw S.H., Chelban V., Turchetti V., Vona B., Tadic V., Houlden H. (2023). Not to Miss: Intronic Variants, Treatment, and Review of the Phenotypic Spectrum in *VPS13D*-Related Disorder. Int. J. Mol. Sci..

[11] Bean B.D., Dziurdzik S.K., Kolehmainen K.L., Fowler C.M., Kwong W.K., Grad L.I., Davey M., Schluter C., Conibear E. (2018). Competitive organelle-specific adaptors recruit Vps13 to membrane contact sites. J. Cell Biol..

[12] Seong E., Insolera R., Dulovic M., Kamsteeg E., Trinh J., Brüggemann N., Sandford E., Li S., Ozel A.B., Li J.Z. (2018). Mutations in *VPS13D* lead to a new recessive ataxia with spasticity and mitochondrial defects. Ann. Neurol..

[13] Richards S., Aziz N., Bale S., Bick D., Das S., Gastier-Foster J., Grody W.W., Hegde M., Lyon E., Spector E. (2015). Standards and guidelines for the interpretation of sequence variants: A joint consensus recommendation of the American College of Medical Genetics and Genomics and the Association for Molecular Pathology. Genet. Med..

